# Contribution of local *Salmonella* infection to autoreactivity: divergent roles of type I interferons in colonization and autoantibody production

**DOI:** 10.1128/mbio.01478-26

**Published:** 2026-08-04

**Authors:** Shingo Bessho, Kaitlyn C. M. Grando, Sophia Olubajo, Anita T. Kowal, Amanda L. Miller, Michael H. Lee, Vincent Tam, Andres J. Klein-Szanto, R. Paul Wilson, Stefania Gallucci, Çağla Tükel

**Affiliations:** 1 Center for Microbiology and Immunology, Lewis Katz School of Medicine, Temple University 12314, Philadelphia, Pennsylvania, USA; 2 Histopathology Facility, Fox Chase Cancer Center 6565, Philadelphia, Pennsylvania, USA; University of California, Berkeley, Berkeley, California, USA

**Keywords:** *Salmonella*, biofilms, curli, type I Interferon, autoimmunity, autoantibody, anti-DNA, amyloid

## Abstract

*Salmonella* biofilm-associated amyloid protein curli is a potent inducer of systemic inflammation and autoimmunity, promoting anti-double-stranded DNA (dsDNA) autoantibodies and pro-inflammatory cytokines, including type I interferons (IFNs). Although type I IFNs are key drivers of autoantibody production in systemic lupus erythematosus (SLE), their role in bacterial biofilm-induced autoimmunity remains unclear. To address this, we utilized type I IFN receptor-deficient (*Ifnar^−/^*^−^) mice. Repeated curli administration induced anti-dsDNA autoantibodies independently of IFNAR signaling. We next orally infected C57BL/6 and *Ifnar^−/^*^−^ mice with a *Salmonella enterica* serovar Typhimurium (STm) Δ*spiB* mutant, which permits long-term colonization without lethal disease. While IFNAR deficiency altered local immune responses and reduced bacterial persistence beginning at 7 days post-infection, it did not affect autoantibody generation. Because extrafollicular splenic responses contribute to both *Salmonella* immunity and antibody-mediated autoimmunity, we examined germinal center activity and splenic involvement. Δ*spiB* infection neither suppressed nor stimulated splenic germinal center formation. Nevertheless, both wild-type and Δ*spiB* infections induced anti-dsDNA autoantibodies, suggesting that autoreactive responses occur independently of germinal center activity. Consistent with this, splenectomized mice infected with Δ*spiB* produced anti-dsDNA autoantibodies at levels comparable to sham-surgery controls. Together, these findings demonstrate that curli- and *Salmonella*-induced autoimmunity can arise independently of both type I IFN signaling and the spleen. Our results further identify early gastrointestinal colonization by *S. Typhimurium* as sufficient to trigger systemic autoreactive responses through mechanisms distinct from classical lupus pathways.

**IMPORTANCE:**

Persistent colonization of the gastrointestinal tract by bacterial pathogens can have long-term consequences for host immunity that extend beyond the site of infection. Here, we show that intestinal colonization by *Salmonella enterica* serovar Typhimurium is sufficient to trigger systemic anti-DNA autoantibody responses, even in the absence of type I interferon signaling or the spleen. While type I interferon signaling promoted bacterial persistence, it was dispensable for autoreactive antibody production, revealing that bacterial colonization and autoimmunity can be uncoupled. Unexpectedly, splenectomized mice generated anti-DNA autoantibodies at levels comparable to controls, demonstrating that the spleen is not required for infection-induced autoreactivity. These findings identify early host-microbe interactions in the gut as a key driver of systemic immune dysregulation and highlight how bacterial colonization can shape immune responses and disease outcomes far beyond the intestinal environment.

## INTRODUCTION

Autoimmune diseases arise when the immune system mistakenly targets self-antigens, leading to inflammation and tissue damage. While genetic predisposition, such as specific HLA genotypes, contributes to susceptibility, environmental factors, particularly infections, play a significant role in disease onset and progression (1, 2). Bacterial infections can either trigger the initial development of autoimmune diseases in genetically susceptible individuals or exacerbate symptoms in those with pre-existing autoimmune conditions. One example is *Salmonella enterica* serovar Typhimurium (STm), an invasive enteric bacterium and a common cause of gastroenteritis worldwide (3). Emerging evidence suggests that STm biofilms and components of the biofilm matrix contribute to the autoimmune responses, potentially acting as persistent immune stimulants (4, 5). STm biofilms are formed throughout the gastrointestinal tract during infection (5, 6). Evidence suggests that once the epithelial barrier is damaged during infection, biofilm-associated molecular patterns (BAMPs) from STm biofilms, particularly the amyloid protein curli, initiate inflammatory responses (4, 5, 7–9). Curli comprises 85% of the extracellular matrix of enteric biofilms and forms complexes with other components of the matrix, such as extracellular DNA (eDNA) and cellulose, providing structural stability to the biofilm (10). Multiple studies have demonstrated that both curli fibers and eDNA act as potent BAMPs recognized by the innate immune system. Curli is detected by Toll-like receptor (TLR) 2/1 on the surface of immune cells, as well as by the cytosolic NLRP3 inflammasome (11, 12). Additionally, eDNA engages TLR9 within the endosomal compartment (13). Indeed, the curli/eDNA complexes elicit a stronger immune response than curli or eDNA alone (14). This heightened immune stimulation is likely due to the complexes simultaneously activating multiple receptors across different cellular compartments. This interaction drives the production of pro-inflammatory cytokines, including IL-6, TNF-α, IL-1β, type I interferons (IFNs), and IL-17 (4, 7, 8, 12–16). Furthermore, mice injected with purified curli naturally complexed with DNA or infected with wild-type (WT) STm, but not curli-deficient mutants, consistently develop anti-double-stranded (ds) DNA autoantibodies, a hallmark of the autoimmune disease lupus (4, 5, 8, 13, 14). Most importantly, the anti-dsDNA autoantibody response can be triggered in both lupus-prone and inbred wild-type mice, suggesting that the curli/DNA complex can contribute to breaking of self-tolerance (14).

Affinity maturation of the B-cell responses commonly occurs in the germinal centers, but such response is inhibited during the early stages of STm infection (17–19). Despite the absence of germinal centers in the spleen, antibodies against *Salmonella* form in extrafollicular sites and are essential, as pre-incubating STm with serum low-affinity antibodies inhibits STm colonization of the spleen just as effectively as serum high-affinity antibodies (17). Extrafollicular B cells have been studied primarily in the spleen, while their presence in mucosal immune sites and their role in responses to *Salmonella* infection remain unknown. Although the spleen generates protective antibodies against STm, it is unclear whether splenic extrafollicular responses contribute to the production of bacteria-induced anti-dsDNA antibodies.

Type I IFN and T_H_17 responses are both critically involved in the pathogenesis of systemic and organ-specific autoimmune diseases, including systemic lupus erythematosus (SLE), systemic sclerosis, primary Sjögren’s syndrome, rheumatoid arthritis, type 1 diabetes, primary biliary cholangitis, and dermatomyositis (20–28). Genetic variants in type I IFN pathway genes are frequently overrepresented in patients with these conditions, highlighting the central role of this pathway in autoimmunity (22). Among these diseases, the role of type I IFNs, particularly IFN-α and IFN-β, is most extensively studied in SLE. SLE is characterized by an “IFN signature,” defined by the elevated expression of type I IFN-stimulated genes (ISGs) in peripheral blood and affected tissues (29, 30). Notably, genetic polymorphisms in key regulators of type I IFN signaling, such as interferon regulatory factor 5 (*IRF5*) and *IRF7*, as well as melanoma differentiation-associated gene 5 (*MDA5*), have been strongly associated with increased susceptibility to SLE (31–33). High serum levels of the IFN signature in SLE patients correlate with disease activity, severity, specific clinical manifestations, and elevated titers of anti-dsDNA autoantibodies (34, 35). These findings have driven the development of targeted therapies aimed at blocking type I IFN signaling. One such treatment, Anifrolumab, a monoclonal antibody targeting the type I IFN receptor, has been approved by the U.S. Food and Drug Administration (FDA) for the treatment of SLE (36). While Anifrolumab is very effective in some patients, like those with cutaneous lupus, the impact of this new drug on the whole population of SLE patients has been underwhelming (37), suggesting that IFNs may play a limited role in lupus pathogenesis.

Type I IFN signaling was initially recognized for its critical role in antiviral defense (38, 39). More recently, it has also garnered attention for its involvement in the immune response to bacterial infections. During *Salmonella* infection, the expression of type I IFN-inducible genes is markedly upregulated. Studies have shown that *Salmonella* can exploit type I IFN signaling to enhance its colonization of the intestinal tract and to impair clearance from the host (40–42). Indeed, this is demonstrated in studies of *Ifnar*^-−/−^ and *Ifn-β*^−/−^ mice, wherein *Salmonella* infections via intravenous, intraperitoneal (i.p.), and intragastric injection are not only better controlled in the early stages, but knockout mice survive significantly longer than wild-type counterparts (42, 43). While type I IFN can play a role in downregulating *Salmonella* flagellin in bone marrow-derived macrophages (44) and promote *Salmonella* clearance via the autophagy pathway (45), the survival of *Ifnar^−/−^* and *Ifn-β^−/−^* mice is attributable to the inhibition of macrophage necroptosis, enhanced IL-1β response, and the lack of STAT2-mediated gut inflammation (42, 43, 46).

As type I IFNs can facilitate acquired humoral immune responses (47) and contribute to autoantibody formation in lupus-prone mice (48–50), we wanted to investigate the role of this pathway during *Salmonella*- and curli-induced autoimmune responses. To do this, we utilized mice deficient in the receptor for IFN-α and IFN-β (IFNAR-deficient, *Ifnar^−/−^* mice) on the C57BL/6J background. Because C57BL/6 mice are susceptible to STm infection, we developed an improved long-term infection model using a STm Δ*spiB* mutant, deficient in a type III secretion system 2, and tested the role of IFN signaling in STm colonization. Prompted by the finding that STm Δ*spiB* mutant induces autoantibodies without reaching the spleen, we also assessed the requirement for the spleen in the *Salmonella*-induced production of anti-dsDNA autoantibody. Our results indicate a role for bacterial infections in triggering a type I IFN-independent autoimmunity and highlight the lack of a role for the spleen in fomenting autoreactivity, which may possibly be played by local, mucosal sites.

## RESULTS

### Curli-induced anti-dsDNA autoantibody production is independent of type I IFNs

**Fig 1:**
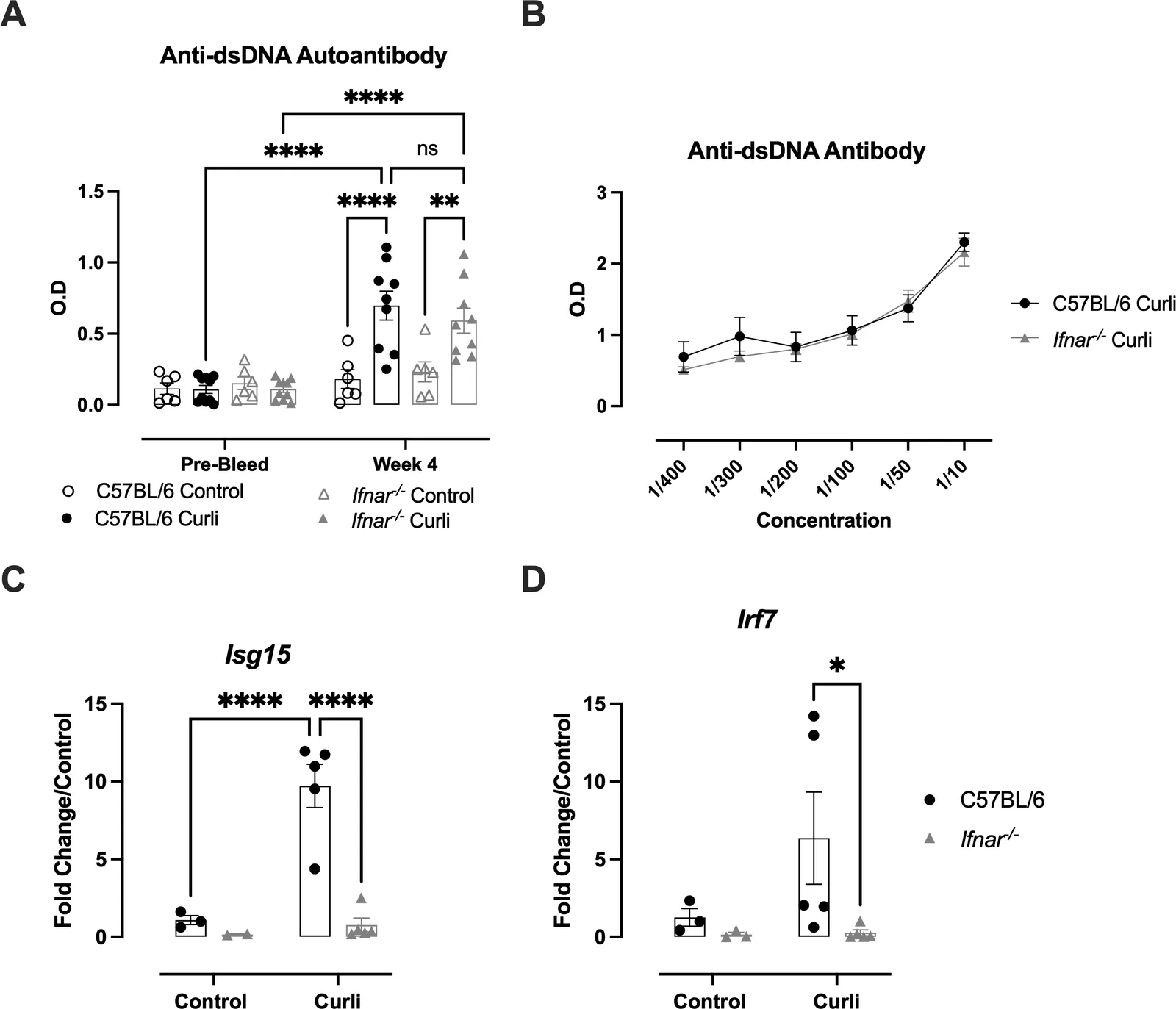
Curli induces anti-dsDNA autoantibodies in an IFNAR-independent manner. (**A**) Levels of anti-dsDNA autoantibodies in the mouse serum, quantified by ELISA from mice i.p. injected with curli or PBS for 4 weeks. (**B**) Levels of anti-dsDNA autoantibodies in the mouse serum for each dilution, quantified by ELISA from mice treated with curli for 4 weeks. Levels of (**C**) *Isg15* and (**D**) *Irf7* were determined by RT-qPCR in RNA extracted from peritoneal lavage fluid of mice 4 h after treatment with curli or PBS. Data were normalized to C57BL/6 mice treated with PBS. Each data point represents one mouse sample. Mean and standard error were calculated by averaging results from two independent experiments. Significance was determined by using a two-way ANOVA with Tukey’s multiple comparison test; *P < 0.05, **P < 0.01, and ****P < 0.0001.

Previous studies have demonstrated that type I IFN expression and anti-dsDNA antibodies can be triggered by purified curli in C57BL/6 mice (4, 5, 8, 13, 14). Here, we first investigated whether the production of autoantibodies in response to systemic curli exposure was dependent on type I IFN signaling. C57BL/6 and *Ifnar^−/−^* mice were i.p. injected with curli twice per week for 4 weeks, using a protocol we have previously established (4, 5, 8, 13, 14). Serum levels of anti-dsDNA autoantibodies were measured via ELISA. We found that curli induced anti-dsDNA autoantibodies in C57BL/6 and *Ifnar^−/−^* mice without significant differences (Fig. 1A). To further evaluate the antibody response, we performed the ELISA for the anti-dsDNA autoantibodies by serially diluting sera from curli-treated mice. The analysis revealed that antibody titers were comparable across groups (Fig. 1B), suggesting that type I interferon signaling has little or no effect on curli-induced autoantibodies. As a functional control, we measured the type I IFN response at the site of injection by collecting the cells in the peritoneal lavage fluid of C57BL/6 and *Ifnar^−/−^* mice 4 h after i.p. injection with curli purified from STm biofilms, and performing qPCR to determine the expression of IFN-stimulated gene 15 (*Isg15*) and *Irf7*. C57BL/6 mice showed significantly elevated expression of both *Isg15* and *Irf7* in response to curli, with little to no response in *Ifnar^−/−^* mice (Fig. 1C and D), confirming that curli induces a type I IFN response in an IFNAR-dependent manner. Overall, these results suggest that while the curli-induced type I IFN response is robust and IFNAR-dependent, it does not play a critical role in the generation of curli-induced anti-dsDNA autoantibodies.

### *Salmonella* SPI-2 mutant (Δ*spiB*) elicits anti-dsDNA autoantibody production in 129Sv/J mice

Next, we wanted to confirm whether type I IFN signaling plays a role in anti-dsDNA autoantibody production in the context of infection. Previous studies into STm infection-induced autoimmune responses were necessarily performed in 129Sv/J and CBA strains because they are resistant to acute STm-induced lethality (51). Unfortunately, C57BL/6 mice, the genetic background of *Ifnar^−/−^* mice, are highly susceptible to STm and typically succumb to infection within 5–8 days (52), making them unsuitable for studying long-term autoimmune sequelae. To examine the role of type I interferons in *Salmonella*-induced autoimmunity, we required a model that permits persistent infection in C57BL/6 mice without rapid mortality, thereby allowing us to observe the development of autoantibodies over time.

**Fig 2:**
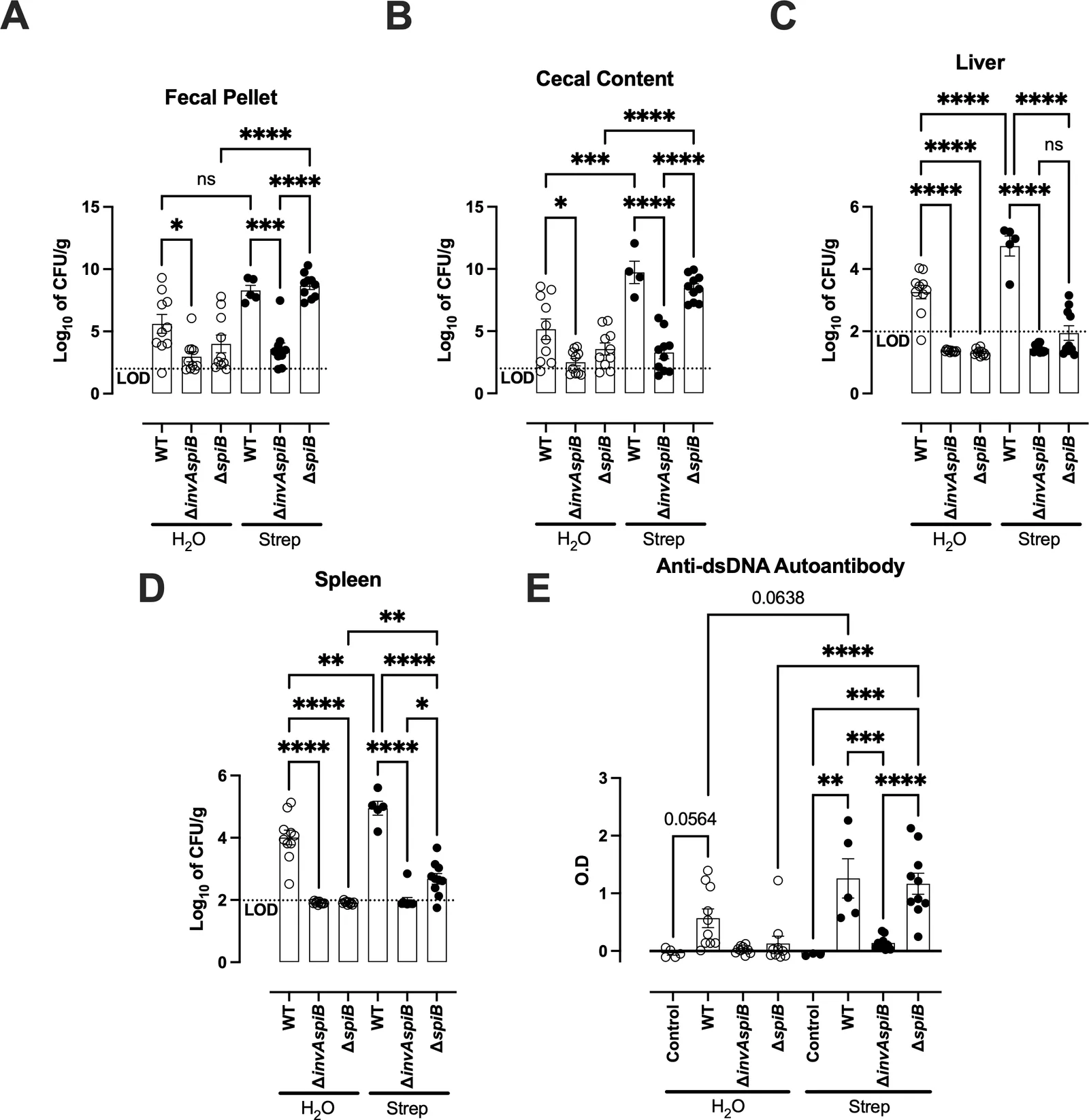
The effect of STm infection on anti-dsDNA autoantibodies of 129/SvJ mice. 129/SvJ mice were pretreated with water or streptomycin and 24 h later infected with 10^4^ CFU of WT, Δ*invAspiB*, Δ*spiB*, or LB control. CFU was measured at 21 dpi in (**A**) fecal pellet, (**B**) cecal content, (**C**) liver, and (**D**) spleen. (**E**) Levels of anti-dsDNA autoantibodies in the mouse serum at 21 dpi, quantified by ELISA. Each data point represents one mouse sample. Mean and standard error were calculated by averaging results from two independent experiments. The dotted line indicates the lower detection limit of CFU plating. Significance was determined by using an ordinary one-way ANOVA with Tukey’s multiple comparisons test; *P < 0.05, **P < 0.01, ***P < 0.001, and ****P < 0.0001.

We previously demonstrated in 129Sv/J and CBA mice that wild-type (WT) STm infection leads to anti-dsDNA autoantibody production. In contrast, no autoantibodies were triggered by a double mutant lacking components of both the SPI-1 and SPI-2 pathogenicity islands (Δ*invAspiB*) (5), which encode two type III secretion systems required for gut barrier invasion and intracellular survival of the pathogen, respectively (53, 54). Unlike the single Δ*invA* mutant, the Δ*spiB* mutant retains the ability to colonize the Peyer’s patches and infect C57BL/6 mice (55). Building on our previous study demonstrating that the Δ*spiB* mutant establishes long-term infection in C57BL/6 mice (7), we sought to advance this model and evaluate its utility as a platform for studying chronic infection and autoimmunity in STm-susceptible mouse strains. To do this, we pretreated 129Sv/J mice with streptomycin or water 24 h before infection. Streptomycin is known to enhance STm gut colonization in mice, and the resulting pathology resembles gastroenteritis (56). After pre-treatment, we intragastrically administered 10^4^ CFU of WT STm, Δ*invAspiB*, or Δ*spiB*. Mice were sacrificed 21 days post-infection (dpi). Bacterial numbers in fecal pellets and cecal contents showed no significant difference in colonization between WT STm and Δ*spiB* (Fig. 2A and B). However, streptomycin pretreated mice had significantly higher levels of both WT STm and the Δ*spiB* mutant in fecal samples throughout the infection (data not shown), and fecal and cecal contents at 21 dpi compared to water pre-treatment controls (Fig. 2A and B). In contrast, the Δ*invAspiB* failed to establish successful colonization based on feces and cecal content (Fig. 2A and B). In the liver and spleen, Δ*spiB* exhibited significantly reduced colonization compared to WT STm (Fig. 2C and D), which was expected given that SPI-2 is essential for *Salmonella* survival and replication in macrophages (57). As anticipated, the Δ*invAspiB* colonization was undetectable in the spleen and liver (Fig. 2C and D).

Importantly, ELISA analysis of anti-dsDNA autoantibody levels revealed that only WT STm induced autoantibody production in water pretreated controls (Fig. 2E). However, when mice were pretreated with streptomycin, there was a significant increase in anti-dsDNA antibody levels following infection with both WT STm and Δ*spiB*. Notably, the levels of anti-dsDNA autoantibodies were similarly elevated in both groups (Fig. 2E). Consistent with previous observations, the Δ*invAspiB* failed to increase anti-dsDNA antibody levels regardless of pre-treatment (Fig. 2E). These findings suggest that the Δ*spiB* may be used as a model for persistent STm infection in C57BL/6 mice, if streptomycin pretreated, enabling the study of *Salmonella*-induced autoimmunity while circumventing rapid lethality.

### Type I IFN deficiency alters *Salmonella* SPI-2 mutant (Δ*spiB*) colonization but not anti-dsDNA autoantibody production

**Fig 3:**
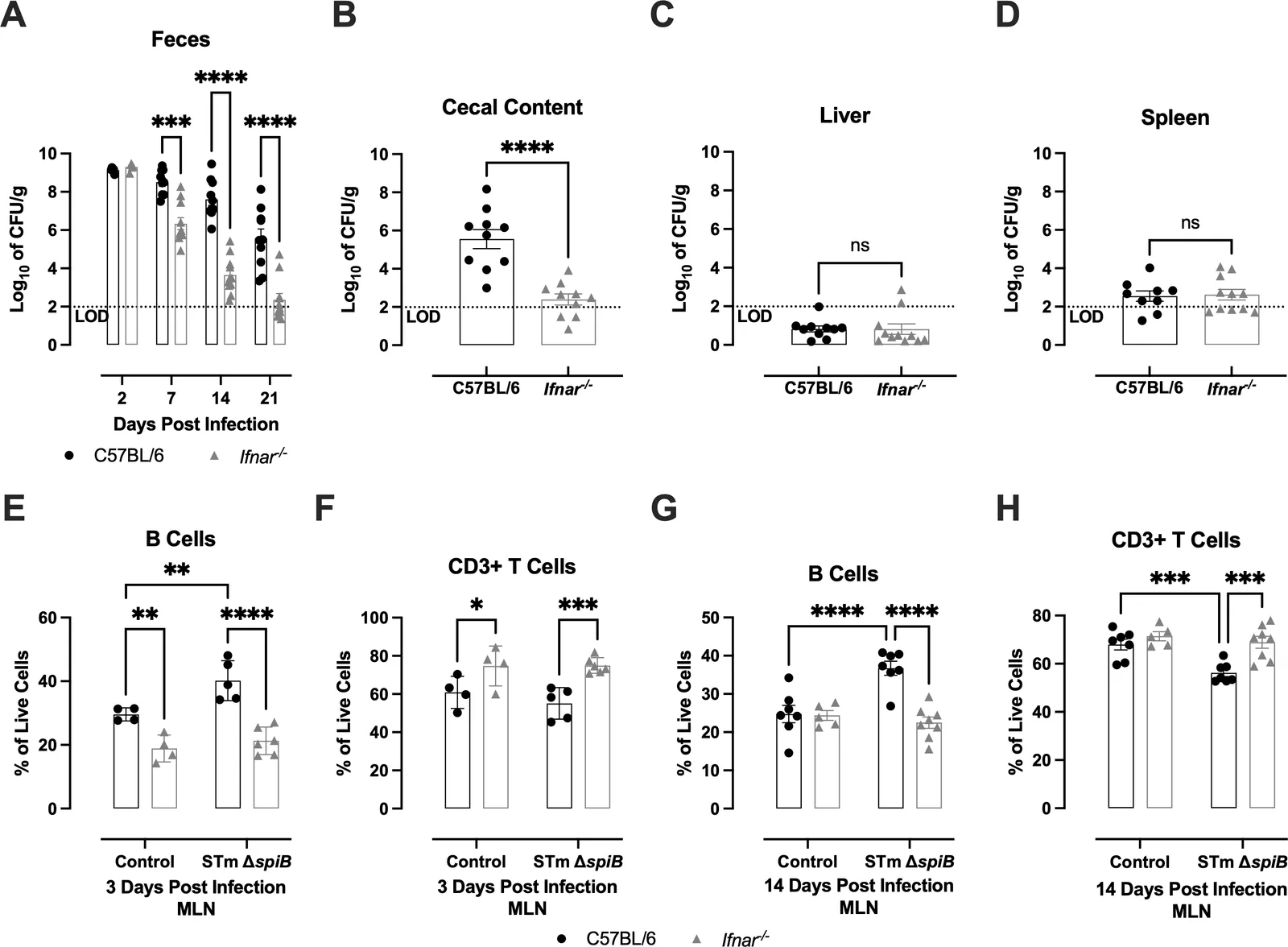
The effect of STm Δ*spiB* infection on MLN immune cells of C57BL/6 and *Ifnar^−/−^* mice. C57BL/6 and *Ifnar^−/−^* mice were pretreated with streptomycin and were uninfected or infected with 10^7^ CFU of STm Δ*spiB*. (**A**) CFU was determined in fecal samples at 2, 7, 14, and 21 dpi. CFU was determined at 21 dpi in (**B**) cecal content, (**C**) liver, and (**D**) spleen. Flow cytometry analysis of MLN at 3 dpi of (**E**) B and (**F**) T cells, and at 14 dpi of (**G**) B and (**H**) T cells. Each data point represents one mouse sample. Mean and standard error were calculated by averaging results from two independent experiments. The dotted line indicates the lower detection limit of CFU plating. Significance was determined by using the Mann-Whitney test and two-way ANOVA with Tukey’s multiple comparisons test; *P < 0.05, **P < 0.01, ***P < 0.001, and ****P < 0.0001.

With the Δ*spiB* model validated, we next infected both C57BL/6 and *Ifnar*^−/−^ mice with 10^7^ CFU of Δ*spiB* following streptomycin pre-treatment. While Δ*spiB* successfully colonized the gastrointestinal tract of both C57BL/6 and *Ifnar^−/−^* mice at 2 dpi, *Ifnar*^−/−^ mice exhibited a significant reduction in Δ*spiB* CFUs starting at 7 dpi, which continued to decline through 21 dpi (Fig. 3A). Reduced Δ*spiB* CFU counts were also observed in the cecal contents at 21 dpi (Fig. 3B). Low levels of bacterial numbers were detected in both liver and spleen of either mouse strain without significant differences (Fig. 3C and D).

**Fig 4:**
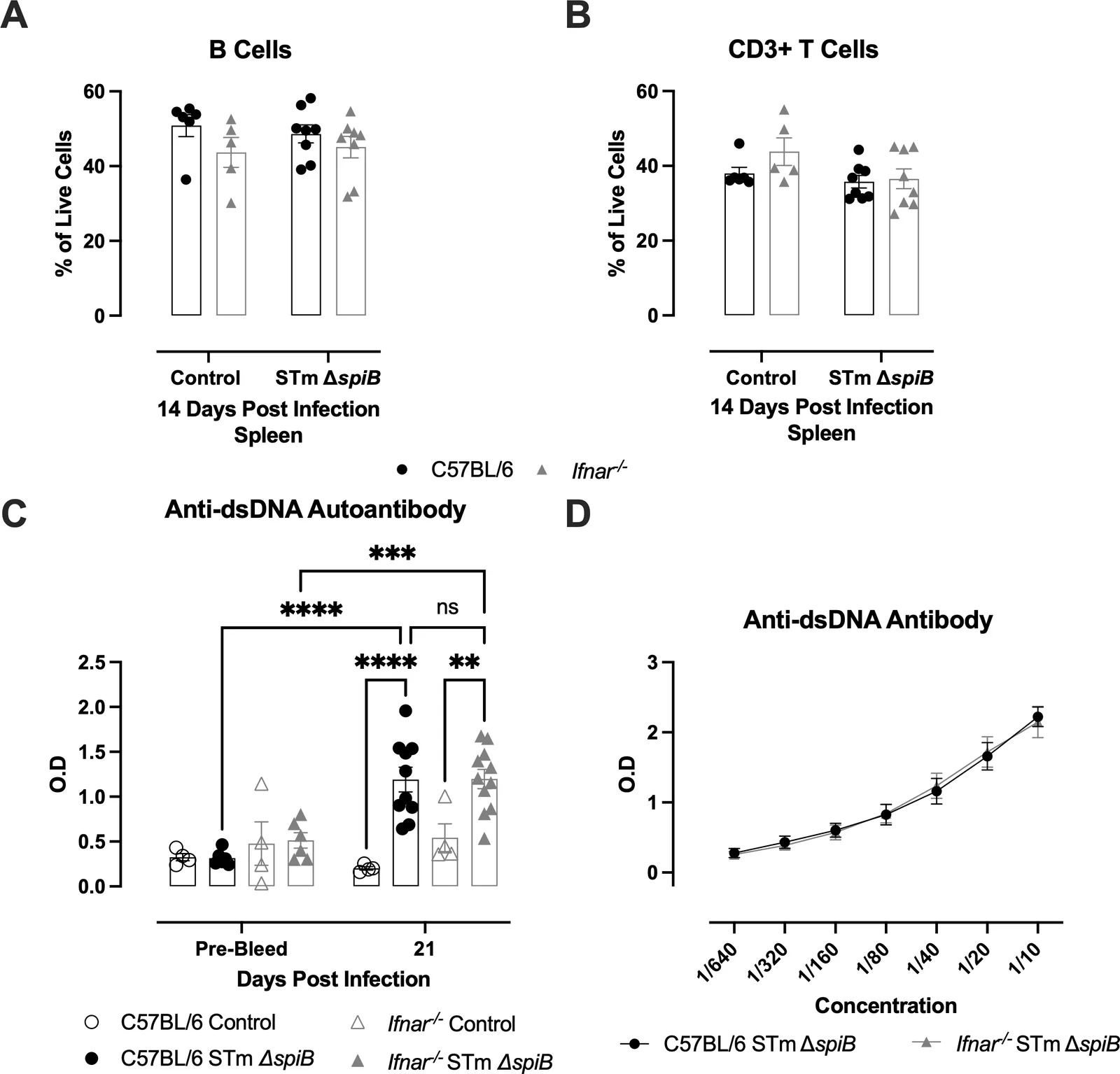
The effect of STm Δ*spiB* infection on splenic immune cells and anti-dsDNA autoantibodies. C57BL/6 and *Ifnar^−/−^* mice were pretreated with streptomycin and were uninfected or infected with 10^7^ CFU of STm Δ*spiB*. Flow cytometry analysis of spleen at 14 dpi of (**A**) B and (**B**) T cells. (**C**) Levels of anti-dsDNA autoantibodies in the mouse serum of pretreated and 21 dpi, quantified by ELISA. (**D**) Levels of anti-dsDNA autoantibodies in the mouse serum for each dilution, quantified by ELISA from infected mice at 21 dpi. Each data point represents one mouse sample. Mean and standard error were calculated by averaging results from two independent experiments. Significance was determined by using two-way ANOVA with Tukey’s multiple comparisons test; **P < 0.01, ***P < 0.001, and ****P < 0.0001.

When the main subsets of lymphocytes were investigated in the mesenteric lymph nodes (MLNs), which partially drain the site of infection, by flow cytometry at 3 and 14 dpi, the B-cell ratio was elevated in infected C57BL/6 mice compared to controls. In contrast, *Ifnar^−/^*^−^ mice showed B-cell ratios similar to the uninfected mice (Fig. 3E and G). In contrast, CD3^+^ T cells appeared slightly lower in C57BL/6 MLNs 3 dpi, though this change did not reach statistical significance, nor was a difference observed with infection in *Ifnar^−/−^* mice (Fig. 3F). However, CD3^+^ T-cell ratios were higher in *Ifnar^−/−^* than in C57BL/6, regardless of infection (Fig. 3F). Significant increases in the percentages of B cells and a decrease in the percentage of T cells in the MLN of infected mice were observed at 14 dpi compared to controls. However, no changes were detected in the *Ifnar^−/−^* mice (Fig. 3G and H). When innate immune cells were analyzed, a significant increase in macrophage recruitment in C57BL/6 was detected in the infected MLNs at 3 dpi, while no differences were observed in *Ifnar^−/−^* mice (Fig. S1A). Other innate immune cells analyzed had no apparent differences (Fig. S1). Despite statistical differences we had observed in B-cell and CD3^+^ T-cell populations in the MLNs (Fig. 3G and H), we observed no significant differences in the ratio percentage of B cells or CD3^+^ T cells in the spleen (Fig. 4A and B), nor plasma cells in either MLNs or spleen (Fig. S2) between mouse genotypes nor treatments at 14 dpi, suggesting that major effects remain at local levels.

Notably, anti-dsDNA autoantibody levels in the serum of both groups of mice were elevated following infection with STm compared to uninfected mice. However, despite lower levels of bacterial load detected in *Ifnar*^−/−^ mice, the levels of anti-dsDNA autoantibodies were similar to the levels of antibodies in STm-infected C57BL/6 mice (Fig. 4C). The titers of the anti-dsDNA autoantibodies were also comparable in a serial dilution assay between the groups (Fig. 4D). Our findings on the anti-dsDNA autoantibody production, from both curli-injected and *Salmonella*-infected mice, suggest that type I IFN signaling is not required for autoantibody production in either context.

### Anti-dsDNA autoantibody production induced by *Salmonella* infection does not require splenic immune cells

**Fig 5:**
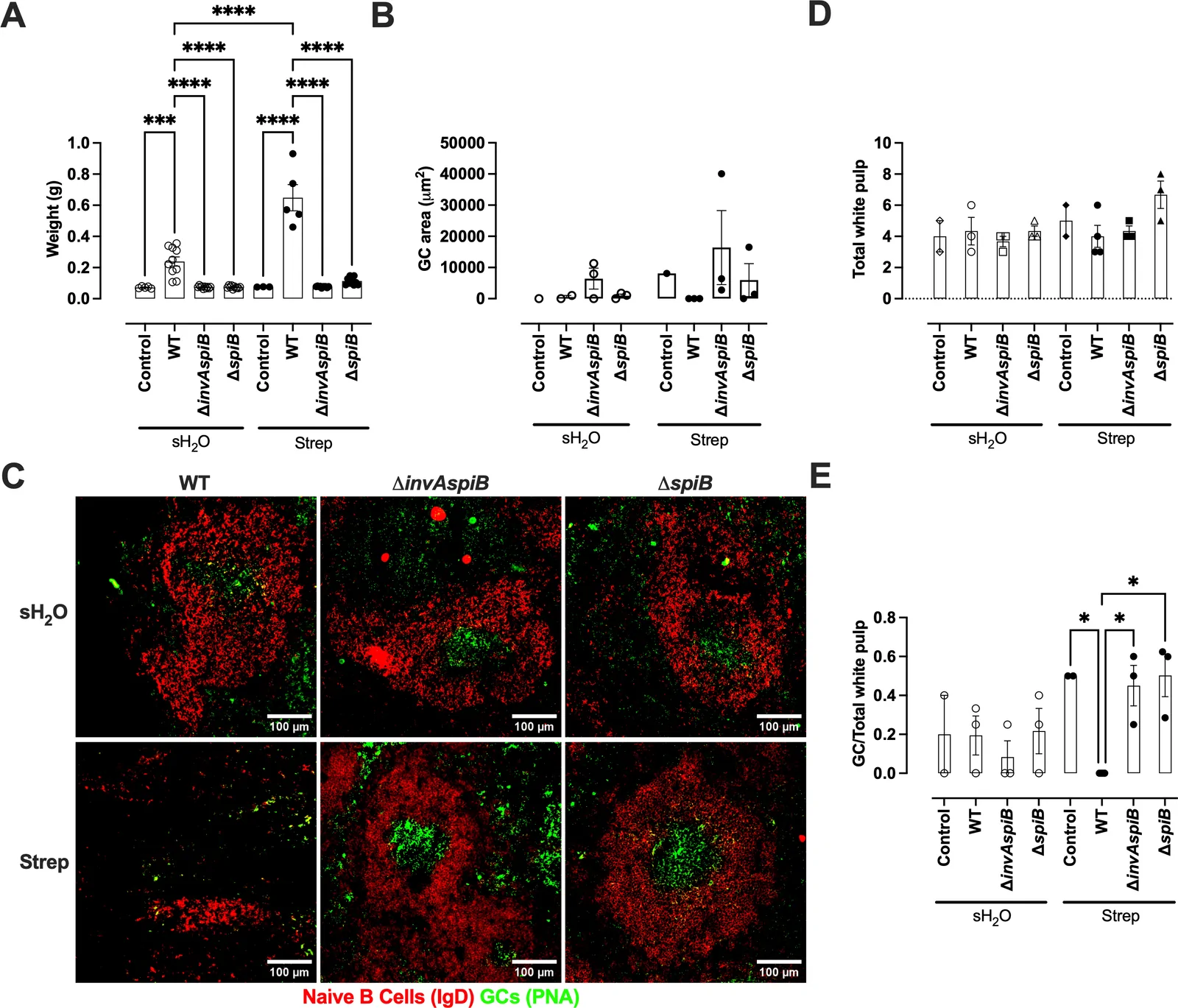
The effect of STm infection on spleen weight and germinal center formation. 129/SvJ mice were pretreated with water or streptomycin and infected with 10^4^ CFU of WT, Δ*invA*, or Δ*spiB* STm. (**A**) Weight of spleen was measured at 21 dpi. (**B and C**) Spleen was fixed and sections were stained with IgD (red) and peanut agglutinin (green). The slides were blinded and evaluated for germinal centers. (**B**) Germinal center area was measured and averaged per mouse. (**C**) Representative images of stained spleens. Hematoxylin and eosin-stained spleen was counted for the total number of white pulp (**D**) and germinal centers within the white pulp (**E**) by a blinded pathologist. Mean and standard error were calculated by averaging results from (**B, D, and E**) one or (**A**) two independent experiments. Significance was determined by using an ordinary one-way ANOVA with Tukey’s multiple comparisons test; *P < 0.05, ***P < 0.001, and ****P < 0.0001.

Germinal centers within the spleen are essential for the development of high-affinity antibodies, memory B cells, and long-lived plasma cells. It was previously reported (17, 18) that WT STm infection increases spleen weight and suppresses the germinal center formation in the spleens of mice. Since no significant differences in the adaptive immune responses were detected in the spleen of C57BL/6 and *Ifnar*^−/−^ mice, we wanted to study the role of splenic immune cells in the production of anti-dsDNA autoantibodies. To study this, we first measured the weights of spleens isolated from 129Sv/J mice pretreated with either water or streptomycin, then infected with WT STm, Δ*invAspiB*, or Δ*spiB* mutants. We observed significantly heavier spleens in mice that were infected with WT STm compared to uninfected, Δ*invAspiB*, or Δ*spiB* mutants, regardless of pre-treatment (Fig. 5A). However, mice prereated with streptomycin had significantly larger spleens after WT STm infection compared to water pre-treatment before WT STm (Fig. 5A), suggesting that the spleen maintains involvement in the host response to infection. It is, however, important to reiterate that, despite significant differences in spleen phenotypes, we observed no differences in anti-dsDNA autoantibody production between mice infected with WT STm and the Δ*spiB* mutant (Fig. 2E).

We further examined this phenotype by assessing germinal center formation in the spleen using peanut agglutinin (PNA) staining. Consistent with previous reports (17, 18), mice infected with WT STm displayed markedly smaller germinal centers than those infected with the Δ*invAspiB*, Δ*spiB*, or the control mice (Fig. 5B and C). A pathologist independently confirmed this observation on hematoxylin- and eosin-stained spleen sections by counting the total number of white pulp (the primary immune regions in the spleen) and then evaluating for germinal centers within the white pulp (Fig. 5D and E). The pathology in streptomycin-pretreated, WT STm-infected mice was characterized as reactive, whereas other groups showed no obvious responses. Notably, although infection with the Δ*spiB* mutant did not increase spleen weight or alter germinal center formation, similar to WT-infected mice, it still elicited anti-dsDNA autoantibody levels comparable to those induced by WT STm. This finding indicates that the development of autoantibody responses can occur independently of overt splenic pathology or germinal center expansion.

**Fig 6:**
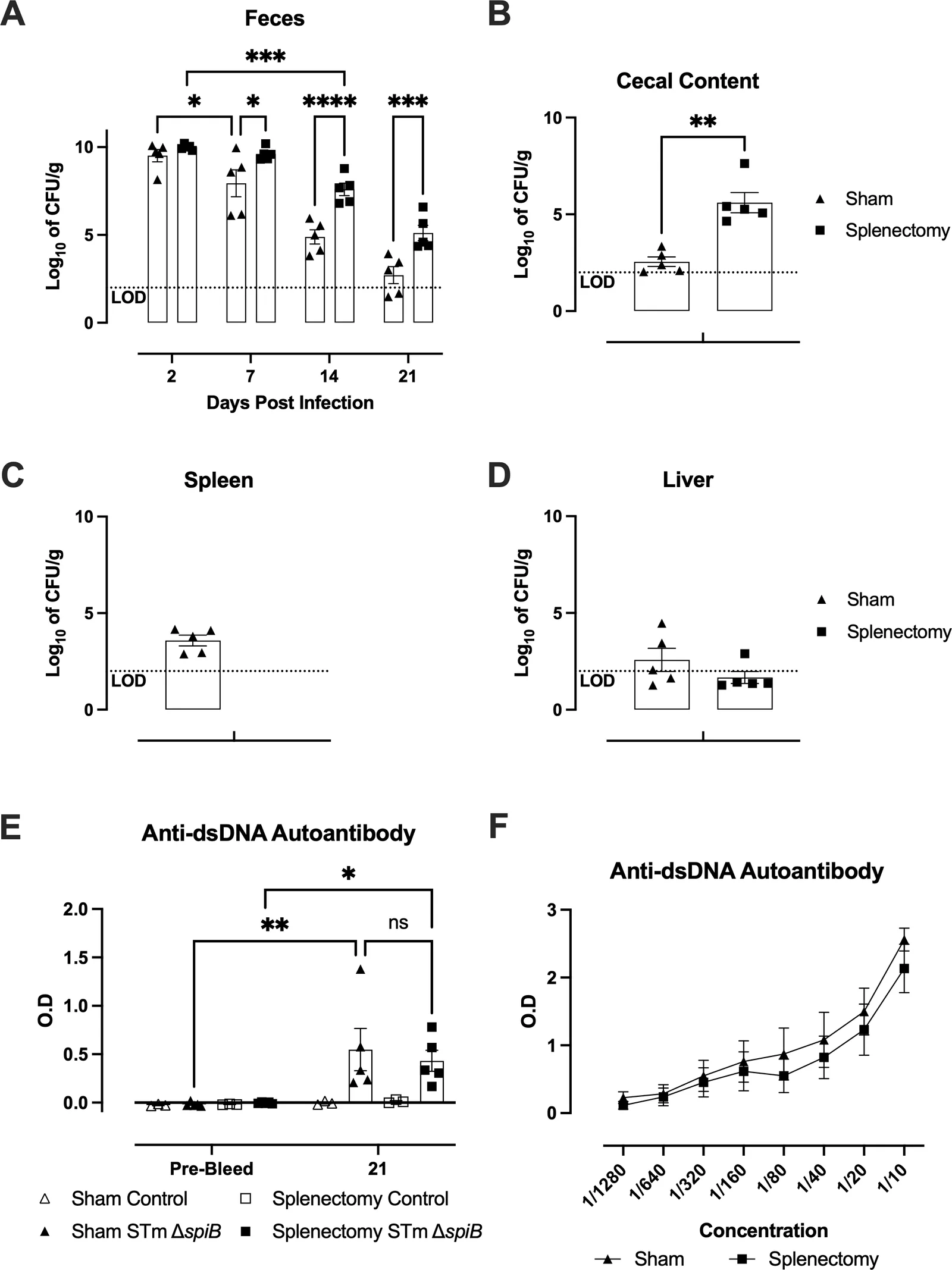
The effect of splenectomy on anti-dsDNA autoantibody production. Splenectomized or sham C57BL/6 mice were pretreated with streptomycin and were infected with 10^7^ CFU of STm Δ*spiB*. (**A**) CFU were determined in fecal samples at 2, 7, 14, and 21 dpi. CFU was determined at 21 dpi in (**B**) cecal content, (**C**) spleen, and (**D**) liver. (**E**) Levels of anti-dsDNA autoantibodies in the mouse serum of pretreated and 21 dpi, quantified by ELISA. (**F**) Levels of anti-dsDNA autoantibodies in the mouse serum for each dilution, quantified by ELISA from infected mice at 21 dpi. Each data point represents one mouse sample. Mean and standard error were calculated by averaging results from one experiment. The dotted line indicates the lower detection limit of CFU plating. Significance was determined by using the Mann-Whitney test and two-way ANOVA with Tukey’s multiple comparisons test; *P < 0.05, **P < 0.01, ***P < 0.001, and ****P < 0.0001.

*Salmonella* has been shown to induce extrafollicular B-cell responses in the spleen (19). To determine the role of the spleen in the generation of anti-DNA responses, we utilized splenectomized C57BL/6 mice and sham surgery (undergone surgery without the removal of the spleen) controls. We infected these mice with 10^7^ CFU of Δ*spiB* after streptomycin pre-treatment. While the Δ*spiB* mutant successfully colonized the gastrointestinal tract of both groups at 2 dpi, mice with the sham surgery exhibited a significant reduction in Δ*spiB* CFUs starting at 7 dpi, while mice lacking spleens did not begin to clear the infection until 14 dpi (Fig. 6A). Both groups continued to clear the infection until the end point, with lower CFU in sham surgery controls compared to the splenectomized mice group (Fig. 6A). Increased Δ*spiB* CFU counts were also observed in the cecal contents of the splenectomized mice compared to sham controls at 21 dpi (Fig. 6B). Low bacterial numbers were detected in the spleens of sham surgery control mice and the livers of either group without significant differences (Fig. 6C and D). Notably, no significant difference was detected in anti-dsDNA autoantibody levels in the serum of mice from both groups (Fig. 6E). The dilutions of the anti-dsDNA autoantibodies were similar between the groups (Fig. 6F). These findings suggest that splenic immune cells are dispensable for the generation of anti-dsDNA autoantibodies during *Salmonella* infection, and that immune activation at the primary sites of infection and local antigen presentation may play a more central role in driving this response.

## DISCUSSION

Bacterial infections can elicit autoimmune responses, including the production of autoantibodies, but the mechanisms that trigger these responses are poorly understood. In mouse models, *Salmonella* infection induces a robust type I IFN response (46) and leads to the production of anti-dsDNA autoantibodies (5, 58), both of which are hallmarks of SLE (59). Our previous work identified that *Salmonella* biofilms, particularly curli–extracellular DNA (eDNA) complex that forms in the extracellular matrix, are key triggers of these autoimmune responses (4, 5, 7, 8, 13, 14). In support of these results in mice, SLE patients who are going through disease flares present elevated levels of anti-curli antibodies, suggesting exposure to curli-producing bacteria (60). Given the established role of type I IFN, especially IFN-α, in promoting anti-dsDNA autoantibodies through dendritic cell maturation, B-cell activation, and differentiation (61–63), we investigated whether type I IFN signaling contributes to STm- and curli-induced anti-dsDNA antibody production. Surprisingly, we found that the absence of type I IFN signaling did not alter anti-dsDNA autoantibody levels following *Salmonella* infection or curli injection (Fig. 1 and 4). These results indicate that the induction of autoantibodies by curli and bacterial infections in mice is type I IFN-independent. If these results can be extrapolated to humans, they imply that autoimmunity initiated or amplified by bacterial infections may develop independently of type I interferon pathways and could, therefore, exhibit limited responsiveness to therapeutic approaches, such as Anifrolumab, that neutralize IFNAR signaling in SLE patients. These results support future clinical studies to determine whether failure of Anifrolumab to improve disease activity in SLE patients is associated with the presence of high levels of anti-curli antibodies and/or signs of recent bacterial infections, an important step in personalizing this therapeutic strategy in SLE.

Consistent with previous reports, we observed that IFN-I signaling restricted *Salmonella* colonization in the gut. Mice lacking the type I IFN receptor, IFNAR, despite being colonized with similar bacterial loads at early time points, showed enhanced clearance of *Salmonella* compared to C57BL/6. Although there were differences in the gut colonization of mice, similar levels of anti-dsDNA antibodies were detected in both C57BL/6 and *Ifnar*^−/−^ mice (Fig. 2). These findings suggest that the comparable anti-dsDNA levels observed in both C57BL/6 and *Ifnar*^−/−^ mice may be due to similar bacterial burdens early in infection, which are sufficient to initiate an autoimmune response. In addition, since C57BL/6 are resistant to arthritis (64), we were unable to evaluate joint inflammation. Nevertheless, type I IFNs may still influence other downstream disease manifestations such as joint inflammation in humans, highlighting their multifaceted role in *Salmonella*-induced autoimmunity.

In this study, we also utilized a novel long-term infection model (7) that allowed us to colonize C57BL/6 without rapid mortality, thereby allowing us to observe the development of autoimmunity over time. To do this, we used a Δ*spiB* mutant that lacks a key SPI-2 component required for intracellular survival, making it an attenuated strain that is rapidly cleared by host immunity and helps dissect the role of intracellular persistence in pathogenesis and autoimmunity. We determined that, unless the mice were pretreated with streptomycin, the Δ*spiB* mutant was unable to induce the anti-dsDNA autoantibodies. However, upon streptomycin pretreatment, which increased the colonization of the Δ*spiB* mutant in the GI tract (Fig. 2A and B), the levels of anti-dsDNA autoantibodies were as elevated as in mice infected with the WT STm (Fig. 2E). These results are important because widespread usage of antibiotics has coincided with a rising incidence of autoimmune diseases (65). While antibiotics help fight and contain infections, their use has a profound impact on our microbiota that is still not completely understood. Depending on the types and lengths of antibiotics used, it may take some members of the microbiome weeks, if not months or years, to recover (66, 67). While it is widely believed that microbiome disruption and the loss of beneficial microbes contribute to the pathogenesis of autoimmune diseases, our findings suggest an alternative or complementary mechanism. Specifically, we speculate that the antibiotic treatment may selectively enrich biofilm-producing bacteria and promote local intestinal inflammation, which in turn may drive the formation of anti-dsDNA autoantibodies.

Our results also showed that the spleen, an important site for antibody production, is not essential for the generation of anti-dsDNA antibodies (Fig. 6). It has been demonstrated that *Salmonella* disrupts the germinal centers following infection (18), and the follicular B cells outside germinal centers play a critical role in the establishment of protective antibody responses to *Salmonella* (19). Similarly, extrafollicular B cells have been shown to be important in spontaneous lupus (68–70). Here, we show that autoimmunity triggered by curli-producing bacterial infections also induces extrafollicular B-cell activation without germinal center formation, thus extending the role for this unconventional B cell maturation in lupus. Moreover, in our study, mice lacking the spleen developed comparable levels of autoantibodies, suggesting that peripheral sites, such as gut-associated lymphoid tissue or other secondary lymphoid organs, may compensate for splenic function or even be the leading site in driving autoantibody responses under inflammatory conditions.

In conclusion, our study shows that autoantibodies induced by bacterial stimuli arise independently of both the spleen and type I interferon signaling, an organ and signaling pathway traditionally considered central to autoantibody generation. We believe that our findings reveal a previously underappreciated mechanism in which bacterial biofilms and local mucosal inflammation are sufficient to initiate systemic autoimmunity. Rather than simply reflecting a loss of beneficial microbes, antibiotic-induced dysbiosis may actively promote the emergence of biofilm-forming pathobionts that drive autoantibody responses. These insights shift the focus from microbial diversity to microbial structure and immune context, opening new avenues for therapeutic intervention in autoimmune disease.

## MATERIALS AND METHODS

### Bacterial strains and growth conditions

Three *Salmonella enterica* serovar Typhimurium strains were used in this study. The wild-type strain, IR715, is a spontaneous nalidixic acid-resistant derivative of the virulent ATCC 14028 isolate and was routinely cultured in Luria-Bertani (LB) broth supplemented with nalidixic acid (50 µg/mL) at 37°C (4, 5). The SPI-1/SPI-2 double mutant (*invA spiB*) and the SPI-2 mutant (*spiB*), previously described (5), were kindly provided by Dr. Andreas Bäumler (University of California, Davis). The *invA spiB* strain was maintained in LB broth containing tetracycline (20 µg/mL) and kanamycin (100 µg/mL), whereas the *spiB* mutant was cultured in LB broth supplemented with kanamycin (100 µg/mL). Unless otherwise indicated, all bacterial cultures were incubated at 37°C.

### Purification of curli

For curli purification, *S. Typhimurium* IR715 Δ*msbB* was cultured in 150 mL YESCA broth supplemented with 4% DMSO in 250 mL flasks as described previously (4). Cultures were incubated for 72 h at 26°C with agitation (200 rpm). The cells were harvested by centrifugation (10,000 rpm, JA-14 rotor, J2-HS Beckman centrifuge) for 10 min at 4°C and resuspended in 10 mM Tris-HCl (pH 8.0). To remove contaminating nucleic acids, suspensions were treated with RNase A (0.1 mg/mL; Sigma, R5502), DNase I (0.1 mg/mL; Sigma, DN25), and MgCl_2_ (1 mM) for 20 min at 37°C. Bacterial lysis was achieved by sonication (30% amplitude, two 30-s pulses), followed by incubation with lysozyme (1 mg/mL; Sigma, L6876) at 37°C for 40 min. SDS was then added to a final concentration of 1%, and samples were incubated for an additional 20 min at 37°C with shaking (200 rpm).

The insoluble curli fraction was recovered by centrifugation, resuspended in 10 mL of 10 mM Tris-HCl (pH 8.0), and boiled for 10 min. A second nuclease treatment with RNase A and DNase I was performed under the same conditions described above to further eliminate residual nucleic acids. Following centrifugation, the curli-containing pellet was washed with 10 mM Tris-HCl (pH 8.0), resuspended in SDS-PAGE sample buffer, and boiled again for 10 min before electrophoretic separation on a 12% resolving gel with a 3–5% stacking gel at a constant current of 20 mA overnight. Because polymerized curli fibers were too large to migrate into the gel, they remained at the top of the gel and were excised, washed three times with sterile water, and extracted twice with 95% ethanol. The purified curli material was finally resuspended in sterile water. Protein concentrations were determined using the BCA assay (Novagen, 71285-3), and all preparations were normalized to a final concentration of 1 mg/mL.

### Treatment of mice

C57BL/6 (wild-type) mice purchased from Jackson Laboratory were bred in our facility. 129 Sv/J mice, splenectomy mice (C57BL/6), and sham surgery mice (C57BL/6) were purchased from Jackson Laboratory at 5–7 weeks of age and were used after acclimating in our facility for 1 week. *Ifnar^−/−^* mice were kindly provided to us by Dr. Stefania Gallucci. At 6–8 weeks of age, mice were injected i.p. with 50 µg of curli/DNA complex or sterile PBS (control) twice a week, alternating sides for 4 weeks. For the 4-h time point, mice were injected once with 100 µg of curli/DNA complex or sterile PBS (control) and sacrificed 4 h later.

For infections, mice were pretreated with streptomycin or water. The streptomycin-pretreated mouse model has been described previously (56). Briefly, 6- to 8-week-old mice were inoculated intragastrically with 20 mg of streptomycin (0.1 mL of a 200 mg/mL solution in sterile water) 24 h before bacterial inoculation. For water-treated mice, instead of streptomycin, they were given an equal volume of sterile water. Bacteria were grown shaking in LB broth with appropriate antibiotics at 37°C overnight. Mice were inoculated intragastrically with either 0.1 mL of sterile LB broth (mock infection) or 10^7^–10^8^ CFU of appropriate bacteria. Mice were euthanized at the indicated time points after infection. Mice were tail bled, and fecal samples were obtained once per week. At the point of sacrifice, blood was obtained from each mouse by cardiac puncture. To determine the number of viable *S*. Typhimurium, samples of liver, spleen, cecal content, and feces were collected from each mouse, homogenized in PBS, and 10-fold serial dilutions were plated on LB agar plates containing appropriate antibiotics. If no colonies were detected in the sample, the value of 0.5 CFU was assigned. Limits of detection were calculated by averaging the CFU per gram of this hypothetical 0.5 CFU.

### Flow cytometry

Immune cells from mesenteric lymph nodes or spleen were isolated and stained as previously described (4). Briefly, isolated organs were dissociated on a 100 µm strainer using the plunger of a 1 mL syringe. Approximately 1 × 10^6^ cells were resuspended in a buffer composed of PBS and 5% FBS. The cells were then resuspended in anti-mouse CD16/CD32 (Fc Shield, Tonbo Biosciences 70-0161-U500). After 15 min at 4°C, the cells were then stained with the antibodies (4), diluted in buffer according to the manufacturer’s instructions. The cells were incubated for 30 min at 4°C in the dark. After staining, the cells were centrifuged and rinsed with FACS buffer, then resuspended in 2% paraformaldehyde for 15 min in the dark at room temperature. Data were collected using a BD FACSymphony A5 and analyzed with FlowJo Software (version 10.10.0). Gating was performed as cited previously (4).

### RNA isolation and qPCR quantification

RNA was isolated from cells using FastRNA Pro Green Kit (MP Biomedical, 116045050) according to the manufacturer’s instructions and as previously described (4). Briefly, 100−300 mg of the sample was transferred to a tube containing Lysing Matrix D and an aliquot of 1,000 µL of RNA Pro solution. The tube was then processed in the FastPrep instrument for 40 s at a setting of 6.0. The sample was then centrifuged at 12,000 rcf for 5 min at 4°C. The supernatant was transferred to a new tube and incubated at room temperature for 5 min. Three hundred microliters of chloroform was added and vortexed for 10 s. The sample was incubated at room temperature for 5 min, then centrifuged at 12,000 rcf for 5 min at 4°C. The upper phase was transferred to a new tube and 500 µL of cold 100% ethanol was added. The sample was then incubated at −20°C for at least 30 min. After centrifugation, the pellet was washed with 500 µL of cold 75% ethanol. The pellet was left to air dry for 5 min at room temperature. The pellet was then resuspended in 50–100  µL of DEPC H_2_O. RNA was quantified using a NanoDrop spectrophotometer.

For each sample, 0.1 µg of RNA per reaction was then reverse transcribed to cDNA using a TaqMan Reverse Transcription kit according to the manufacturer’s protocol (Invitrogen, N8080234). qPCR was performed on Applied Biosystems StepOne Plus Real-Time PCR system as described previously (8). Transcript levels were determined using the ∆CT approach. Primer sequences were listed previously (46).

### Analysis of anti-dsDNA autoantibodies by ELISA

ELISAs were performed according to a previously published protocol (71) and as previously described (4). Briefly, a 96-well plate (Costar, 07-200-33) was coated with 0.01% poly-l-lysine (Sigma, P8920) in PBS and incubated at room temperature for 1 h. Plates were then washed three times with distilled water and coated with 2.5 µg/mL of calf thymus DNA (Invitrogen, 15633-019) in borate-buffered saline (BBS) (17.5 g NaCl, 2.5 g H_3_BO_3_, and 38.1 g sodium borate in 1 L H_2_O) and incubated overnight at 4°C. The next day, plates are washed three times with BBS and blocked with 200 µL/well of BBT (BBS with 3% bovine serum albumin and 1% Tween 20) for 2 h at room temperature with gentle rocking. After washing five times with BBS, serial dilutions of control serum and sample sera were incubated on the plate overnight at 4°C. Next, plates were washed three to five times with BBS, and biotinylated goat anti-mouse IgG (Jackson ImmunoResearch, 115-065-071) was added. Samples were incubated at room temperature for 2 h with gentle rocking.

After five washes, serial dilutions of control serum and sample sera were incubated on the plate overnight at 4°C. Next, plates were washed three times and incubated with biotinylated goat anti-mouse IgG (Jackson ImmunoResearch, 115-065-071) at room temperature for 2 h. After five washes, the plates were incubated with Avidin-alkaline phosphate conjugate (Sigma, A7294) for 2 h at room temperature. Plates were washed five times and incubated with 1 mg/mL of 4-nitrophenyl phosphate disodium salt hexahydrate (Sigma-Aldrich, N2765) at 37°C protected from light for 2–3 h. Optical densities at 650 and 405 nm were determined using a Molecular Devices Microplate reader. Positive control sera were taken from MRL-lpr lupus-prone mice with high levels of autoantibodies and used at a dilution of 1:250 in BBT.

For the antibody avidity assay, the ELISA protocol was followed, but the serum samples were diluted as indicated in BBT.

### Evaluation of germinal centers

Following infection with STm, the spleen was extracted. For immunofluorescence, spleens were frozen in Optimal Cutting Temperature compound over dry ice. Sections (5 µm thick) were obtained using Leica CM1860. Sections were left to dry at room temperature for 10 min, then fixed in acetone for 5 min at −20°C. Slides were air-dried at room temperature for 10 min, then rehydrated in staining buffer (1% bovine serum albumin and 1% Fc block in PBS) for 10 min. Slides were then labeled with peanut agglutinin (FL-1071-10; Vector Laboratories) and PE anti-mouse IgD (11-26c.2a; BioLegend 405705) at 1:100 concentration in staining buffer in the dark for 60 min at room temperature. After washing with PBS, slides were incubated with anti-rat Rhodamine RedX secondary antibody (Jackson ImmunoResearch) at 1:100 concentration in staining buffer in the dark for 60 min at room temperature. After washing with PBS, slides were mounted in VectaShield (H-1000-10; Vector Laboratories), imaged on the EVOS FL Auto 2, and the area of the germinal center was manually quantified with ImageJ (National Institute of Health).

For hematoxylin and eosin staining, the spleen was fixed in 10% phosphate-buffered formalin for 48 h. Samples were then dehydrated in 70% ethanol before they were embedded in paraffin. Sections of 5 μm of the tissue were stained with hematoxylin and eosin. The fixed and stained sections were blinded and evaluated by an experienced pathologist. Histopathological analyses were done at the Histopathology core at Fox Chase Cancer Center. White pulp and germinal centers were counted for every sample. For analysis, the number of germinal centers was normalized to the number of white pulps, taking the spleen size into account.

### Statistical analysis

Data were analyzed using Prism software (GraphPad 10.6.1). One-way or two-way ANOVA, as well as Mann-Whitney tests, were used when appropriate. The error was determined by the standard error of the mean. *P* values of <0.05 were considered significant. *P < 0.05, **P < 0.01, ***P < 0.001, and ****P < 0.0001 were marked in the figures.

## Data Availability

All data are fully available without restriction in the supplemental materials.

## SUPPLEMENTAL MATERIAL

Supplemental figures mbio.01478-26-s0001.docx Fig. S1 and S2.

ASM does not own the copyrights to Supplemental Material that may be linked to, or accessed through, an article. The authors have granted ASM a non-exclusive, world-wide license to publish the Supplemental Material files. Please contact the corresponding author directly for reuse.
