# Supplementary material for: Contribution of local Salmonella infection to autoreactivity: divergent roles of type I interferons in colonization and autoantibody production
Source: mBio. 2026 Aug 4;17(9):e01478-26. doi: 10.1128/mbio.01478-26 (PMC13556195; doi:10.1128/mbio.01478-26)
Supplement: Supplementary file 1 [file mbio.01478-26-s0001.docx]

**Supplementary Figures**

**Supplementary Figure 1. The effect of Δ*spiB* infection on the innate immune cells in the MLN of C57BL/6 and *Ifnar^-/-^* mice.** C57BL/6 and *Ifnar^-/-^* mice were pre-treated with streptomycin and were uninfected or infected with 10^7^ CFU of STm Δ*spiB****.*** Flow cytometry analysis of mesenteric lymph nodes at 3 dpi of **(A)** macrophages, **(B)** monocytes, **(C)** neutrophils, and **(D)** conventional dendritic cells. Each data point represents one mouse sample. Mean and standard error were calculated by averaging results from two experiments. Significance was determined by using 2-way ANOVA with Tukey’s multiple comparisons test; *p<0.05.

 **Supplementary Figure 2. The effect of Δ*spiB* infection on plasma cells in the MLN and spleen of C57BL/6 and *Ifnar^-/-^* mice.** C57BL/6 and *Ifnar^-/-^* mice were pre-treated with streptomycin and were uninfected or infected with 10^7^ CFU of STm Δ*spiB****.*** Flow cytometry analysis of plasma cells at 14 dpi of **(A)** MLN and **(B)** spleen. Each data point represents one mouse sample. Mean and standard error were calculated by averaging results from two experiments. Significance was determined by using 2-way ANOVA with Tukey’s multiple comparisons test.
